# Interprofessional In Situ Simulation’s Impact on Healthcare Personnel’s Competence and Reported Need for Training in Cardiopulmonary Resuscitation—A Pilot Study in Norway

**DOI:** 10.3390/healthcare12192010

**Published:** 2024-10-09

**Authors:** Kristina Grasto, Ann-Chatrin Linqvist Leonardsen

**Affiliations:** 1 Faculty of Health, Welfare and Organization, Østfold University College, P.O. Box 700, 1757 Halden, Norway; kristina.grasto@hiof.no; 2 Department of Anesthesia, Østfold Hospital Trust, P.O. Box 300, 1714 Grålum, Norway

**Keywords:** cardiopulmonary resuscitation, competence, in situ simulation, interprofessional

## Abstract

Background/objectives: International guidelines recommend cardiopulmonary resuscitation [CPR] training every sixth month. However, research indicates that more training is needed to maintain CPR competence. The objectives of this pilot study were (a) to assess health personnel’s self-reported competence and need for more training in CPR before and after interprofessional in situ CPR simulation and (b) to assess time since the last CPR course and respondent’s reported need for more training. Also, we wanted a pilot to assess areas of improvement in a future, larger study. Methods: A questionnaire was administered to healthcare personnel in hospital wards receiving CPR training using a purposeful sampling strategy. Results: In total, 311 respondents answered the pre-intervention and 45 respondents answered the post-intervention survey. The respondents believed they had good knowledge, skills, and training in CPR, and about 2/3 of the respondents reported a need for more knowledge, skills, and training. There was a weak positive correlation between the time since the last CPR course and the perceived need for more training [*p* < 0.05]. There were no significant differences in self-reported competence or perceived need for more training pre- and post-intervention. The pilot detected several limitations that need improvement in a future study. Conclusions: The authors suggest that regular training is important for maintaining competence in CPR. Also, in a future study, comparisons at an individual level, as well as assessments by experts and of non-technical skills, should be included.

## 1. Introduction

In 2022, 1235 patients received cardiopulmonary resuscitation [CPR] in Norwegian hospitals. Of these, approximately 30 percent of the patients were alive 30 days after the incident [1]. The «chain of survival» is essential to save lives, including recognition of cardiac arrest and activation of the emergency response system, early CPR, rapid defibrillation, advanced resuscitation, post-cardiac arrest care, and recovery [2]. The quality of CPR has been addressed in several studies [3,4,5], For example, Vestergaard et al. [6] found a significant association between the frequency of chest compressions and three-year survival after cardiac arrest. Studies also underline the importance of training to achieve or maintain competence in CPR [7,8]. International recommendations state that hospitals should have dedicated CPR teams responding to cardiac arrests [7]. Also, the time interval from when the cardiac arrest is recognized until rhythm analysis/the first shock has been delivered should not extend to three minutes [9]. This means that all in-hospital personnel potentially recognizing a cardiac arrest must have the competence needed to initiate and ensure quality CPR. The European Resuscitation Council (ERC) recommends that all healthcare personnel attend CPR courses and maintain their certification, including participating in simulation and learning CPR non-technical skills (NTS), use of cognitive aids, and application of data-driven performance-focused debriefing [10]. 

Several studies have assessed healthcare personnel’s skills in CPR. For example, Lund-Kordahl, Mathiassen [11] identified a positive correlation between healthcare personnel’s self-reported skills in CPR and the actual performance when tested. Also, a significant improvement in chest compressions during training was seen. Similarly, Anderson, Sebaldt [12] found skill improvements in personnel training on a monthly basis, in contrast to those training every third, sixth, or twelfth month [12]. Hardeland et al. [13] explored the impact of either simulation training, tabletop exercise, or an e-learning course on healthcare personnel’s self-reported competence and reported the need for more training in CPR in pregnant women. Even if the reported need for training was significantly reduced after the intervention, 78 percent of the respondents reported still needing training. No significant differences between training methods were identified.

Simulation has been shown to be effective in increasing CPR competence [14]. Systematic reviews have also shown the effect of simulation [15] and interprofessional team training [16] on maintaining in-hospital CPR competence. Simulation is an acknowledged method for learning skills and maintaining or achieving competencies as a supplement to real-life experiences [17,18,19]. Full-scale simulations are resource-demanding. However, in situ simulation, which takes place in the actual working environment and involves those who work there, has been shown to be suitable for interprofessional training in emergency situations [20,21]. However, studies have not focused on the effects of in situ CPR simulations on participants’ CPR competence. 

In Norway, there has been a focus on increasing CPR competence and quality both in the public, in community healthcare services, and in hospitals [22,23]. International guidelines recommend training every six months [2]. However, research indicates that more training is needed to maintain CPR competence [12,13]. We have not identified any studies exploring the effect of in situ CPR simulations on personnel’s self-reported competence and reported the need for more training. 

### Objectives

The objectives of this pilot study were a] to assess healthcare personnel’s self-reported competence and need for more training in CPR before and after an interprofessional in situ simulation of in-hospital cardiac arrest, and b] to assess a potential correlation between time since the last CPR course and respondent’s self-reported need for more training. Also, we aimed to assess the study design and methodology before planning a larger study. 

## 2. Materials and Methods

The pilot study had a quasi-experimental, pre/post-intervention design without randomization [24], using a questionnaire before and after an intervention to assess healthcare personnel’s competence and need for training in-hospital CPR. The study adheres to strengthening the reporting of observational studies in epidemiology (STROBE) [25]. 

### 2.1. Setting 

The pilot was conducted at an emergency hospital in Southeastern Norway, with a catchment area of approximately 320,000 inhabitants. The hospital has over 7000 employees and is divided into surgical, medical, gynecological/obstetric, mental health/addiction, and policlinic departments. There are eleven somatic wards and nine wards within mental health and addiction. In addition, there are specialized wards within intensive care, surgery, post-anesthesia care, medical surveillance, neonatal, maternity, laboratory, and imaging, as well as emergency departments. The hospital has an interprofessional CPR team, which is activated through a calling system. The team comprises professionals with heterogeneous competence and consists of a chief medical officer, a registrar medical officer, a registrar anesthetist, a nurse anesthetist, a doctor on duty, and a nurse from the emergency department. 

### 2.2. In Situ Simulation

Realistic cardiac arrest scenarios were developed together with subject managers from different wards, a CPR coordinator, a CPR registrar officer, and the first author (nurse anesthetist). Resusci Anne Simulator^®^, enabling defibrillation, intubation, and intravenous access, was used. Employees at the pilot wards were informed about the simulation through e-mail; however, they were not informed which day this would be. Hospital CPR guidelines and procedures were attached. Participants alerted the CPR team as usual. However, a “Training” sign was placed in the ward. Facilitators were dressed in yellow vests to indicate their role. Debriefs were conducted after all simulations, focusing on the CPR algorithm, teamwork, and roles of each participant. In total, 60 min were dedicated to the training and debriefing. The in situ simulations were conducted in January and February 2024. 

### 2.3. Sample

The pre-intervention survey included all clinical personnel receiving CPR training within the hospital, using a purposeful sampling strategy. Invitations to participate in the survey were sent to all managers (N = 171), who were requested to distribute this further to their employees. Hence, the population receiving the invitation was unknown. Personnel included radiographs/radiologists, bioengineers, health secretaries, nurse assistants, pediatric nurse assistants, functional disability nurses, registered nurses, specialist nurses, midwives, registrar and consultant physicians, ergotherapists, physiotherapists, and psychologists. 

The participating wards in the pilot in situ simulations were also purposefully selected [24] based on capacity to facilitate the simulations, available personnel, and bed occupancy allowing for simulation. Managers in five wards were requested; however, one of the wards was canceled due to high bed occupancy. 

### 2.4. Data Collection

A previously validated questionnaire, the Competence in Cardiac Arrest in Pregnancy (ComCA-P) [26], was adjusted to the pilot study purpose by the two authors [one nurse anesthetist and one professor experienced with developing questionnaires, both female] together with the hospital’s CPR council members, including registrar medical officers, anesthesiologists, and pediatricians (N = 6). Then, the questionnaire was piloted by eight healthcare professionals, who confirmed the face and content validity of the new questionnaire.

Initially, the respondents were asked to report their ward of employment and professional background. This was followed by seven questions about self-reported education, training, and experience with CPR, as well as their professional role in CPR situations. The adjusted ComCA-P included 12 questions on self-reported competence in CPR, with response alternatives on a 5-point Likert scale from 1 = totally disagree to 5 = totally agree. Questions included self-reported competence [knowledge and skills], need for more competence [knowledge, skills, training], and their assessment of self- and team efficacy in CPR situations. There were also four knowledge questions regarding CPR procedures and a free-text question with the possibility to elaborate on any other issues.

The questionnaire was distributed to the respondents through the University of Oslo’s secure digital platform “Nettskjema” [27]. The pre-intervention survey was conducted in the period October to December 2023. The post-intervention survey was conducted in the pilot wards only in February 2024.

### 2.5. Analysis

Data were analyzed using the Statistical Package for the Social Sciences, version 27. Descriptive statistics and frequencies were used, and data were presented as n (%). Continuous data were tested for normality using Kolmogorov–Smirnov statistics (*p* < 0.05) [28], indicating that data were not normally distributed. Hence, data are presented as medians and interquartile range (IQR). Spearman’s rho was used to assess correlations between variables [27]. Values < 0.2 are assumed weak, 0.3–0.5 average and >0.05 strong correlation [29]. Mann–Whitney U test was used to compare responses pre- and post-intervention. Cronbach’s alpha was used to assess the internal consistency of the questionnaire in the current study. No method for calculating missing values was used. *p* values < 0.05 were assumed statistically significant.

### 2.6. Ethical Aspects

The study was conducted in line with the Helsinki Declaration [30] and based on confidentiality and willingness and informed consent to participate through completed and submitted questionnaire. According to Norwegian legislation, there is no need for ethical approval in studies, including healthcare personnel only. The study was assessed and approved by the Norwegian knowledge sector’s service provider (Sikt, project no. 359859), as well as by the hospital’s data protection officer and the hospital director.

## 3. Results

In total, 311 healthcare personnel responded to the pre-intervention, and 45 responded to the post-intervention questionnaire. Table 1 presents the respondent’s ward, professional background, and CPR education, training, and experience pre-intervention.

Due to the in situ simulations being conducted in four wards in the pilot, the respondent’s background information post-intervention is not presented due to confidentiality issues. Table 1 shows that most respondents were employed in clinical wards, and over 70 percent were nurses. Even if over 80 percent had participated in a CPR course the last year, the median time since the last CPR course was six months. Half of the respondents had simulated CPR during the last 12 months.

### 3.1. Competence and Need for Training Pre- and Post-Simulation

Table 2 presents the respondent’s self-reported CPR competence and need for training before and after the in situ simulation.

Table 2 shows that more than 80 percent of the respondents were assessed to have good knowledge, skills, and training in CPR both before and after the in situ simulation. In the pre-test, 71.4 percent of the respondents reported feeling safe on the CPR algorithm in cardiac arrest situations, compared to 57.8 percent in the post-test. There was also an increased number of respondents reporting needing more knowledge, skills, and training after the intervention (55.6, 59.1, and 68.8, respectively), than before (43.3, 46.9, and 59.8 percent, respectively). However, these differences were not significant. Most respondents reported knowing the warning routines and where to find the necessary equipment; however, the results show that there is room for improvement regarding working together as a team, role clarification, drug administration, and facilitation of CPR training. There were no statistically significant differences between responses pre- and post-intervention beyond how to complete the cardiac arrest registration form, which improved post-simulation.

### 3.2. Correlations

Table 3 presents the results from Spearman’s rho analysis of “time since last CPR course in months” and the respondent’s self-reported need for more training.

The correlation analysis showed a weak, positive, significant correlation between time from last CPR course and the self-reported need for more training. Hence, the need for more training seemed to increase with increasing time from the last course.

## 4. Discussion

Overall, the results from this pilot study indicate that healthcare personnel report to have good knowledge, skills, and training in CPR both before and after in situ simulations. However, they also report a need for more knowledge, skills, and training independent of simulation. Also, the self-reported need for more training seemed to increase with time from the last CPR course. The results also uncovered a self-reported need for team training, role clarification, drug administration, and documentation in CPR, regardless of simulations.

Most of the pre-intervention respondents reported having good knowledge (87.8%), skills (75.9%), and training (88.1%). This contrasts Hardeland, Svendsen [13], where 54 percent of the pre-intervention respondents reported low competence, and 82 percent reported needing more training in CPR in pregnancy. Of course, this may be due to the patient group, namely pregnant women. However, Silverplats, Södersved Källestedt [31] found that healthcare personnel’s knowledge about CPR in general is low and decreasing in line with time since the last CPR course. Also, Bentley et al. [32] identified low knowledge about the use of technical equipment such as defibrillators in CPR simulations. This may again lead to the defibrillator not being used [33,34] and, hereby, low-quality CPR.

In this pilot study, pre-intervention respondents reported having a need for more knowledge (43.3%), skills (46.9%), and training (59.8%), which aligns with the findings from Hardeland et al. [13]. Surprisingly, this increased after the in situ simulations (55.6%, 59.1%, and 68.8%, respectively), even if this was not significant. In addition, we could not identify any significant changes in reported knowledge, skills, training, or need for these, respectively, before and after the intervention. Both these findings contrast previous studies exploring knowledge and skills before and after simulations [12,13,15]. We have no reasonable explanation for this issue other than that the participants may have become aware of their own shortages in knowledge, skills, and training during the in situ simulation. The post-simulation debrief focused on the CPR algorithm, teamwork, and roles of each CPR participant. However, the participants did not get a chance to improve their performance based on the feedback. Hence, the results may have been different if the simulation was repeated. Also, only 45 CPR participants responded to the post-simulation questionnaire. Further, this is in contrast to the observed positive correlation between time since last CPR course and the reported need for more training in CPR, which aligns more with previous research [12,13].

Over 90 percent of the respondents reported knowing the warning routines, namely, how to alert the CPR team and where to find the necessary equipment; however, the results show that there is room for improvement in knowledge and skills regarding working together as a team, role clarification, and drug administration. Several studies underline the positive impact of interprofessional simulation-based training on team learning and patient outcomes [16,35,36]. As such, it is a recommended method by the World Health Organization for emergency preparedness [37]. Our results may suggest that healthcare personnel did not experience increased knowledge and skills and still reported needing training. However, this was a pilot study, and the results may have been different in a larger sample and when compared at an individual level.

The members of the CPR team hold a certification in advanced CPR, including drug administration and airway management. These comprise the registrar, consultant physicians, and some of the specialized nurses. However, most of the participants were not members of the CPR team and held a certification in basic CPR. Since not comparing at an individual level, participants with basic CPR competence may only have reported an increased need for more knowledge and skills due to not having the advanced CPR competence as the members of the CPR team. However, we were not able to assess such associations.

Also, we included questions about the respondents’ assessment of teamwork and role, which is part of the NTS that ERC suggests included as a focus in CPR simulations [10]. As such, some studies have found a positive association between NTS and simulation [19,38,39]. In future studies, assessment of NTS, such as situation awareness, communication, or teamwork before and after CPR simulation, and also both self and expert assessment would be appropriate.

### Limitations

The main limitation of this study is the limited sample size in the post-intervention survey, and the results are not generalizable. However, it was a pilot study, and we can conclude that in situ simulations are feasible in our hospital. Moreover, the responses were not compared at an individual level. We used self-reporting as a tool to measure the outcome of the in situ simulations. This has been criticized in several studies, even if it is an acknowledged method [40]. Boet et al. have summarized various tools to assess team performance in crisis situations [41], and we could have considered using one of these in the current study.

## 5. Conclusions

Healthcare personnel reported good knowledge, skills, and training in CPR both before and after in situ CPR simulations. At the same time, they reported needing more knowledge, skills, and training. The results indicate areas that need to be emphasized in further courses or simulations, such as team organization, role distribution, and drug administration in CPR. However, the pilot provided central information about requirements for future studies, including the opportunity to compare pre- and post-results at an individual level, focus on NTS, and both self and expert assessments.

### Implications

Repeated CPR training is required. This pilot suggests that the participants could benefit from two simulations, allowing for direct improvement based on the debrief. Focusing on the various roles of the CPR participants and how critical skills such as drug and airway management may be limited to those with advanced CPR competence seems essential. The main aim must be to improve and maintain healthcare personnel’s competence in basic CPR and defibrillation.

## Figures and Tables

**Table 1:** Respondent’s characteristics pre-intervention (N = 311).

Employment	*n* (%)
Somatic ward	90 (28.9)
Specialized ward	66 (1.3)
Emergency ward	36 (11.6)
Policlinics	52 (16.7)
Other	67 (21.5)
Professional background	
Nurse assistant	26 (8.5)
Registered nurse	116 (37.3)
Specialized nurse	106 (34.1)
Registrar physician	2 (0.6)
Consultant physician	6 (1.9)
Health secretary	8 (2.6)
Bioengineer	2 (0.6)
Functional disability nurse	1 (0.3)
Other	44 (14.1)
CPR course last 12 months (m = 1)	
Yes	258 (83.2)
No	49 (15.8)
Cannot remember	3 (1)
Months since last CPR course	
Median (IQR)	6 (2–11)
CPR course last 12 months (m = 1)	
Yes	258 (83.2)
No	49 (15.8)
Cannot remember	3 (1)
Months since last CPR course	
Median [IQR]	6 (2–11)
Simulated CPR last 12 months	
Yes	146 (46.9)
No	156 (50.2)
Cannot remember	9 (2.9)
Experienced cardiac arrest last 12 months (m = 2)	
Yes	124 (40.1)
No	180 (58.4)
Cannot remember	5 (1.6)
Number of cardiac arrests experienced the last 12 months	
Median (IQR)	1 (0–2)

CPR = cardiopulmonary resuscitation. IQR = interquartile range. Specialized wards = intensive care, surgery, post-anesthesia care, medical surveillance, neonatal, maternity, laboratory, and imaging. Specialized nurse = nurses with further education/master’s degree.

**Table 2:** Respondent’s self-reported CPR competence and need for training before and after the in situ simulation (N = 311/45).

	Pre-Simulation			Post-Simulation			*p*-Value
	Disagree n (%)	Neither/nor n (%)	Agree n (%)	Disagree n (%)	Neither/nor n (%)	Agree n (%)	
I have good knowledge about CPR (n = 310/45)	5 (1.6)	33 (10.6)	272 (87.8)	2 (4.4)	4 (8.9)	39 (86.7)	0.45
I have good skills in CPR (n = 310/45)	13 (4.1)	62 (20)	235 (75.9)	2 (4.4)	6 (13.3)	37 (82.3)	0.82
I have received good training in CPR at work (n = 310/45)	18 (5.8)	19 (6.1)	273 (88.1)	2 (4.4)	3 (6.7)	40 (88.9)	0.29
I feel safe on the CPR algorithm in cardiac arrest situations (n = 308/45)	35 (11.4)	53 (17.2)	220 (71.4)	4 (8.9)	15 (33.3)	26 (57.8)	0.24
I need more CPR knowledge (n = 309/45)	70 (22.7)	105 (34)	134 (43.3)	5 (11.1)	15 (33.3)	25 (55.6)	0.20
I need more CPR skills (n = 309/44)	71 (23)	93 (30.1)	145 (46.9)	4 (9)	14 (31.9)	26 (59.1)	0.20
I need more CPR training (n = 309/45)	52 (16.9)	72 (23.3)	185 (59.8)	7 (15.6)	7 (15.6)	31 (68.8)	0.91
In CPR we work good together as a team (n = 305/45)	6 (1.9)	105 (34.4)	194 (63.7)	2 (4.4)	17 (37.8)	26 (57.8)	0.47
In CPR, my role is clear to me (n = 309/45)	33 (10.6)	64 (20.7)	212 (68.7)	3 (6.7)	9 (20)	33 (73.3)	0.92
I know the warning routines in CPR (n = 307/43)	9 (2.9)	14 (4.6)	284 (92.5)	1 (2.3)	2 (4.7)	40 (93)	0.19
I know where to find necessary equipment in CPR (n = 310/45)	12 (3.9)	16 (5.2)	282 (90.9)	1 (2.2)	2 (4.4)	42 (93.3)	0.38
I have knowledge about relevant drugs to be used in CPR (n = 307/43)	79 (25.8)	49 (16)	179 (58.2)	5 (11.6)	8 (18.6)	30 (69.8)	0.19
I know how to complete the cardiac arrest registration form (306/44)	192 (62.8)	42 (13.7)	72 (23.5)	19 (43.2)	5 (11.4)	20 (45.4)	0.005 *
My leader facilitates for me to develop and maintain my CPR competence (n = 308/44)	25 (8.1)	68 (22.1)	215 (69.8)	1 (2.3)	7 (15.9)	36 (81.8)	0.27

Response alternatives totally disagree and disagree collated to “disagree”. Response alternatives agree and totally agree collated to “agree”. Number of respondents at each question pre/post-simulation reported in parenthesis. CPR = cardiopulmonary resuscitation. * = statistically significant at a 0.05 level. Mann–Whitney U test.

**Table 3:** Correlation between time since last CPR course and the need for more training (n = 311).

	Totally Disagree	Disagree	Neither Disagree nor Agree	Agree	Totally Agree
Need more CPR training n (%)	11 (3.5)	42 (13.5)	75 (24.1)	136 (43.7)	47 (15.2)
Months since last CPR course Median	1	6	4	6	7.5

Spearman’s rho test. *p* = 0.003.

## Data Availability

Data are available upon request to the last author.
